# Experimental Removal and Recovery of Subtidal Grazers Highlights the Importance of Functional Redundancy and Temporal Context

**DOI:** 10.1371/journal.pone.0078969

**Published:** 2013-11-08

**Authors:** Robin Elahi, Kenneth P. Sebens

**Affiliations:** 1 Friday Harbor Laboratories, University of Washington, Friday Harbor, Washington, United States of America; 2 Department of Biology, University of Washington, Seattle, Washington, United States of America; 3 School of Aquatic and Fisheries Sciences, University of Washington, Seattle, Washington, United States of America; University College Dublin, Ireland

## Abstract

The extent to which different grazers are functionally redundant has strong implications for the maintenance of community structure and function. Grazing by red urchins (*Strongylocentrotus franciscanus*) on temperate rocky reefs can initiate a switch from invertebrate or macroalgal dominance to an algal crust state, but can also cause increases in the density of molluscan mesograzers. In this study, we tested the hypothesis that red urchins and lined chitons (*Tonicella* spp.) are redundant in the maintenance of available space, defined as encrusting algae and bare rock. In a factorial field experiment replicated at three sites, we reduced the densities of urchins and chitons on subtidal rock walls for nine months. The effects of grazers were interpreted in the context of natural temporal variation by monitoring the benthic community one year before, during, and after grazer removal. The removal of each grazer in isolation had no effect on the epilithic community, but the removal of both grazers caused an increase in sessile invertebrates. The increase was due primarily to clonal ascidians, which displayed a large (∼75%) relative increase in response to the removal of both grazers. However, the observed non-additive responses to grazer removal were temporary and smaller than seasonal fluctuations. Our data demonstrate that urchins and chitons can be redundant in the maintenance of available space, and highlight the value of drawing conclusions from experimental manipulations within an extended temporal context.

## Introduction

Grazing is a fundamental biotic process controlling the structure and function of communities. Different species of grazers can compete for the same resource and thus serve a common functional role in a community. Such redundancy among species can be considered a form of ‘biological insurance’, especially in the context of a fluctuating environment [1]. In an age of accelerating climatic fluctuations [2] and global biodiversity loss [3], it is important understand the extent of grazer redundancy in the context of natural temporal variability [4].

Sea urchins are ecologically important grazers in nearshore benthic communities, and can mediate shifts between kelp forests and urchin barrens on temperate reefs [5]–[7]. The kelp state may resist urchin grazing through stabilizing feedbacks such as physical deterrence (e.g., whiplash) [8] and the provision of habitat for the predators of urchin recruits [9]. However, when urchins successfully remove macroalgae from reefs, the indirect effects of grazing can persist after the departure of urchins. For example, increased sedimentation in urchin barrens prevents the recruitment of macroalgae and thus reinforces the persistence of a barrens state in the absence of urchins [10]. In addition, urchins facilitate chitons and other molluscan mesograzers [11], [12], whose grazing represents a candidate stabilizing mechanism for the barrens state.

In this study, we tested the hypothesis that red urchins (S*trongylocentrotus franciscanus*) and lined chitons (*Tonicella* spp.) are redundant in the maintenance of available space, defined here as encrusting algae and bare rock [12]. Chitons are common mesograzers on hard rocky substrata in nearshore marine environments, and like urchins, are capable of structuring benthic communities [13]. Through grazing, these consumers restart succession on benthic rocky substrata by making space for recruitment available, a limiting resource for sessile taxa [14]. Urchins, due to their larger size, are capable of consuming larger macroalgae and sessile invertebrates and thus indirectly facilitate lined chitons by establishing a favorable foraging environment of biofilms and encrusting algae [12]. However, once foraging space is available to chitons, the potential for resource overlap between these grazers exists because both can scrape biofilms and the microscopic recruits of algae and invertebrates from rocky substrata [15]–[17]. The extent to which these grazers compete or complement one another in the maintenance of available space, which relies on the removal of recruits and juveniles of sessile taxa, is unclear.

The roles of urchin and chiton grazing have most often been studied in the context of macroalgal communities [18]–[20], but these omnivorous grazers can also influence the distribution and abundance of sessile invertebrates [21]–[24]. We studied these two grazers on subtidal rock walls, which are dominated typically by epifaunal invertebrates, rather than macroalgae, due to light limitation [25]–[28]. In a factorial field experiment replicated at three sites, we reduced the densities of urchins and chitons for nine months. The effects of grazers were interpreted in the context of natural temporal variation by monitoring the benthic community one year before, during, and after grazer removal.

## Methods

### Field Experiment

In December 2007 (prior to the removal of grazers), permanent transects (2.5 m long, 2 m wide, *n* = 6 site^−1^) and quadrats (0.09 m^2^, *n* = 4 transect^−1^) were established on subtidal rock walls (12–18 m depth) at three sites in the San Juan Islands, Washington, United States of America [12]. Using a split-plot factorial design, we removed urchins from transects (whole-plot factor), and removed chitons from quadrats (within-plot factor). The two grazers were manipulated at different scales because red urchins are larger, less abundant, and more mobile, than lined chitons. Consequently, the split-plot design allowed a test of the effects of urchin removal on chiton abundance, but not chiton removal on urchin abundance.

At each of three sites, urchins were removed from three transects, and three other transects served as controls. The six permanent transects at each site were arranged linearly and parallel to shore, and for the purposes of urchin removal, adjacent transects were paired (to stratify the removal treatments throughout the site). For each pair of transects, the removal treatment was assigned to the transect with higher urchin density (quantified from six surveys between December 2007 and March 2009). Within each transect, the quadrats with the highest and third highest density of chitons (quantified from three surveys between December 2007 and March 2009) were assigned to removal treatments. The remaining two quadrats were not manipulated. The systematic method by which we targeted higher densities of grazers ensured that the treatments were meaningful (i.e., so that removal treatments were, on average, actually removing grazers), but not completely biased (i.e., control treatments did experience some grazing pressure). We acknowledge that our removal methods were unconventional, but they were meant to achieve a middle ground, between random and targeted entirely towards the highest densities. In principle, our methods are similar to removal (or addition) experiments that purposefully target areas of high grazer density for comparison with areas that lack grazers. Consequently, inferences drawn from the latter design (and our design) must be limited to situations in which there are high densities of grazers. Initial field experiments are often designed to determine if there is any potential effect at high (but relevant) population densities, before designing experiments that test the more subtle effects of lower densities. In the following paragraphs (see Analysis), we will highlight potential biases in our response variables that may have arisen due to our design.

Grazer removals began on 18 April 2009 and continued every two weeks until 24 January 2010. The density of urchins within two meters of the 2.5 m transects was quantified prior to their removal. Chiton density was quantified from photographs of quadrats (n = 10 during the experimental period) taken prior to their removal; quadrats were not photographed after each removal. Logistical difficulties associated with winter SCUBA diving in the San Juan Islands prevented the removal treatments from continuing through March, one year after the initial photographs. However, urchin densities were quantified in February 2010. Although experimental treatments were maintained actively for nine months, we considered the experimental period to be one year, from March 2009 to March 2010, and the recovery period to be from March 2010 to March 2011. We deemed this appropriate because these communities exhibit strong seasonality, and furthermore, we expected there to be a lag in the response of the sessile community to the experimental treatments. All of the fieldwork described in this study was conducted within the San Juan County and Cypress Island Marine Biological Preserve, Washington State with permission from the Director of the University of Washington Friday Harbor Laboratories.

### Analysis

To quantify temporal variation in sessile community composition, we analyzed the percent cover in eleven photographs of each permanent quadrat taken between 29 March 2008 and 14 March 2011. The percent cover of sessile organisms was estimated visually from photographs, using a method developed by [29] and modified by [12]. Taxa were scored only if they were attached to rock or encrusting algae, i.e., epibiotic taxa do not occupy primary space and thus were not quantified. We defined available space as the substratum available for the recruitment and growth of macroalgae and sessile invertebrates [30], which included bare rock, calcified encrusting algae, and non-calcified encrusting algae. Encrusting algae are included in the definition of available space because there is very little bare rock in shallow hard-bottom subtidal habitats, and most invertebrates can overgrow coralline and non-calcified algal crusts [31]. In so doing we assumed that these algal crusts are functionally equivalent, in part for simplicity, but also because the extent to which various species of encrusting algae facilitate [32] or inhibit [33] the settlement of other sessile taxa is poorly understood in this community.

Linear mixed effects models and a model selection approach were used to address the effectiveness of experimental treatments and the primary hypotheses. First, we tested the effects of removal treatment on grazer densities (log-transformed) during the experimental period (March 2009– March 2010). Second, we tested the effects of grazer removal on temporal variation in invertebrate and macroalgal cover during the experimental period. Third, we hypothesized that grazer removal would result in an increase in clonal ascidians and a concurrent decrease in the cover of available space.

We studied temporal variation in the sessile community before the experiment (28 March 2008–20 March 2009), during the experiment (21 March 2009–22 March 2010), and during one year of recovery after the experiment (23 March 2010–14 March 2011). Specifically, we quantified the percent cover of sessile invertebrates and macroalgae in quadrats. The comparison of sessile epifauna and macroalgae was of interest because urchins and chitons are studied most often in a macroalgal context [18], [19], and because the abundance of these two sessile functional groups is strongly dependent on the orientation of the rock surface [36]. By targeting higher densities of grazers for removal, it could be argued that our approach led to a reduced chance of detecting a treatment effect in the absolute cover of invertebrates or macroalgae, and was thus a conservative design. This is because control quadrats and transects harbored fewer grazers to begin with, and thus the average difference in grazing pressure between control and removal treatments was lower than if replicates for removal had been selected at random. We compared the set of 19 nested, ecologically relevant models for the data collected during the experiment (March 2009–March 2010). All models included site, transect and quadrat as random effects; quadrat at a specific time point was the unit of replication in this analysis. The fixed effects varied between the models, and these details are listed for each model description (Tables 1, 2, S1 and S2). In all models, time was treated as a categorical factor because the dependent variables (e.g., grazer density, percent cover) were not expected to vary linearly through time (for example, due to seasonality). The percent cover of sessile invertebrates was not transformed, but we used a logit transformation for algal cover to correct the non-linearity observed in a diagnostic plot of residuals against fitted values.

**Table 1:** Results of linear mixed effects models testing the fixed effects of urchin removal (U), chiton removal (C), and time (T) on invertebrate and macroalgal cover during the experimental period (March 2009–March 2010).

Model	*K*	AIC_c_	Δi	*w*_i_	logLik
*Invertebrate cover*					
**y∼U×C×T (saturated model)**	**24**	**2724.23**	**0**	**0.54**	−**1336.32**
y∼U+C+T+U:C+U:T+C:T	20	2728.89	4.66	0.05	−1343.21
**y∼U+C+T+U:C+U:T**	**16**	**2725.73**	**1.51**	**0.25**	−**1346.07**
y∼U+C+T+U:C+C:T	16	2770.36	46.14	0	−1368.39
y∼U+C+T+U:T+C:T	19	2730.56	6.33	0.02	−1345.16
y∼U+C+T+U:C	12	2766.5	42.27	0	−1370.8
y∼U+C+T+U:T	15	2727.46	3.23	0.11	−1348.03
y∼U+C+T+C:T	15	2772.09	47.86	0	−1370.34
y∼U+C+U:C	8	2931.77	207.55	0	−1457.68
y∼U+T+U:T	19	2730.56	6.33	0.02	−1345.16
y∼C+T+C:T	14	2770.09	45.86	0	−1370.43
y∼U+C+T	11	2768.27	44.05	0	−1372.76
y∼U+C	7	2933.59	209.37	0	−1459.64
y∼U+T	10	2767.61	43.38	0	−1373.49
y∼C+T	10	2766.32	42.1	0	−1372.85
y∼U	6	2932.98	208.75	0	−1460.37
y∼C	6	2931.69	207.47	0	−1459.73
y∼T	9	2765.67	41.45	0	−1373.58
y∼1 (null model)	5	2931.09	206.86	0	−1460.46
*Macroalgal cover (logit)*					
y∼U×C×T (saturated model)	24	1456.78	12.17	0	−702.6
y∼U+C+T+U:C+U:T+C:T	20	1456.08	11.47	0	−706.8
y∼U+C+T+U:C+U:T	16	1450.14	5.54	0.02	−708.28
y∼U+C+T+U:C+C:T	16	1451.73	7.13	0.01	−709.07
y∼U+C+T+U:T+C:T	19	1457.46	12.85	0	−708.61
**y∼U+C+T+U:C**	**12**	**1445.96**	**1.35**	**0.17**	−**710.53**
y∼U+C+T+U:T	15	1451.58	6.97	0.01	−710.09
y∼U+C+T+C:T	15	1453.17	8.56	0	−710.89
y∼U+C+U:C	8	1548.03	103.42	0	−765.81
y∼U+T+U:T	14	1450.55	5.94	0.02	−710.67
y∼C+T+C:T	14	1451.24	6.64	0.01	−711.01
y∼U+C+T	11	1447.44	2.84	0.08	−712.34
y∼U+C	7	1549.56	104.96	0	−767.62
**y∼U+T**	**10**	**1446.46**	**1.86**	**0.13**	−**712.92**
**y∼C+T**	**10**	**1445.57**	**0.97**	**0.21**	−**712.47**
y∼U	6	1548.63	104.02	0	−768.2
y∼C	6	1547.74	103.13	0	−767.75
**y∼T**	**9**	**1444.61**	**0**	**0.33**	−**713.05**
y∼1 (null model)	5	1546.82	102.21	0	−768.32

*K = *number of parameters; AIC_c_ = corrected AIC (AIC_c_); Δi = difference in AIC_c_ between the candidate model and the best model; *w*_i_ = Akaike weights; logLik = the log-likelihood (logLik). Candidate models with Δi <2 are listed in bold.

**Table 2:** Results of linear mixed effects models testing the fixed effects of urchin (U) and chiton (C) removal on the change in cover of clonal ascidians and available space after the experimental period (March 2010), and one year of recovery after the experimental period (March 2011).

Model	*K*	AICc	Δi	logLik	*w*_i_
*Experiment; Change* *in clonal ascidians*					
**y∼U×C** **(saturated model)**	**9**	**544.00**	**0.00**	**−261.55**	**0.81**
y∼U+C	8	549.53	5.53	−265.62	0.05
y∼U	7	550.10	6.10	−267.18	0.04
y∼C	7	549.35	5.35	−266.80	0.06
y∼1 (null model)	6	549.99	5.99	−268.35	0.04
*Experiment; Change* *in available space*					
**y∼U×C** **(saturated model)**	**9**	**578.33**	**0.00**	−**278.71**	**0.44**
y∼U+C	8	580.65	2.32	−281.18	0.14
y∼U	7	579.93	1.60	−282.09	0.20
y∼C	7	581.45	3.12	−282.85	0.09
y∼1 (null model)	6	580.80	2.47	−283.75	0.13
*Recovery; Change* *in clonal ascidians*					
**y∼U×C** **(saturated model)**	**9**	**551.78**	**0.00**	−**265.44**	**0.90**
y∼U+C	8	558.44	6.66	−270.08	0.03
y∼U	7	560.16	8.38	−272.21	0.01
y∼C	7	558.26	6.48	−271.25	0.04
y∼1 (null model)	6	560.02	8.25	−273.37	0.01
*Recovery; Change* *in available space*					
y∼U×C (saturated model)	9	566.67	3.55	−272.88	0.06
y∼U+C	8	565.37	2.25	−273.54	0.12
y∼U	7	564.48	1.36	−274.36	0.19
y∼C	7	563.94	0.82	−274.10	0.25
**y∼1** **(null model)**	**6**	**563.12**	**0.00**	−**274.91**	**0.38**

*K = *number of parameters; AIC_c_ = corrected AIC (AIC_c_); Δi = difference in AIC_c_ between the candidate model and the best model; *w*_i_ = Akaike weights; logLik = the log-likelihood (logLik). Candidate models with Δi <2 are listed in bold.

To address the hypothesis that the removal of grazers would change the cover of clonal ascidians and concurrently affect the amount of available space, we quantified annual changes in these functional groups during (March 2009–March 2010; experiment) and after (March 2010–March 2011; recovery) the experimental treatment. By targeting higher densities of grazers for removal, we would expect a larger relative change in the benthic community and in this case our experimental approach is biased towards the detection of an effect. All statistical models included site and transect as random effects; quadrat was the unit of replication in this analysis.

Model fit based on maximum likelihood scores was compared using the small sample unbiased Akaike information criteria (AIC_c_), a metric that considers both model fit and complexity (i.e., number of parameters, K). The difference in AIC_c_ (Δi) between each model and the best model (i.e., lowest AIC_c_) was calculated to emphasize the most plausible models given the data (Δi <2). Finally, Akaike weight (*w*_i_), or the relative likelihood of each model, was obtained by normalizing the likelihood across the entire set of candidate models. Details on model selection are provided in Burnham and Anderson [34] and Johnson and Omland [35]. Residuals were inspected visually for normality and homoscedasticity. When necessary, dependent variables were log or logit transformed. Maximum likelihood was used to estimate parameters in all mixed effects models. Statistical analyses were conducted using the packages ‘lme4’ [37] and ‘vegan’ [38] in R 2.14 [39]. Data used in this manuscript are publicly available as Dataset S1.

## Results

**Figure 1:**
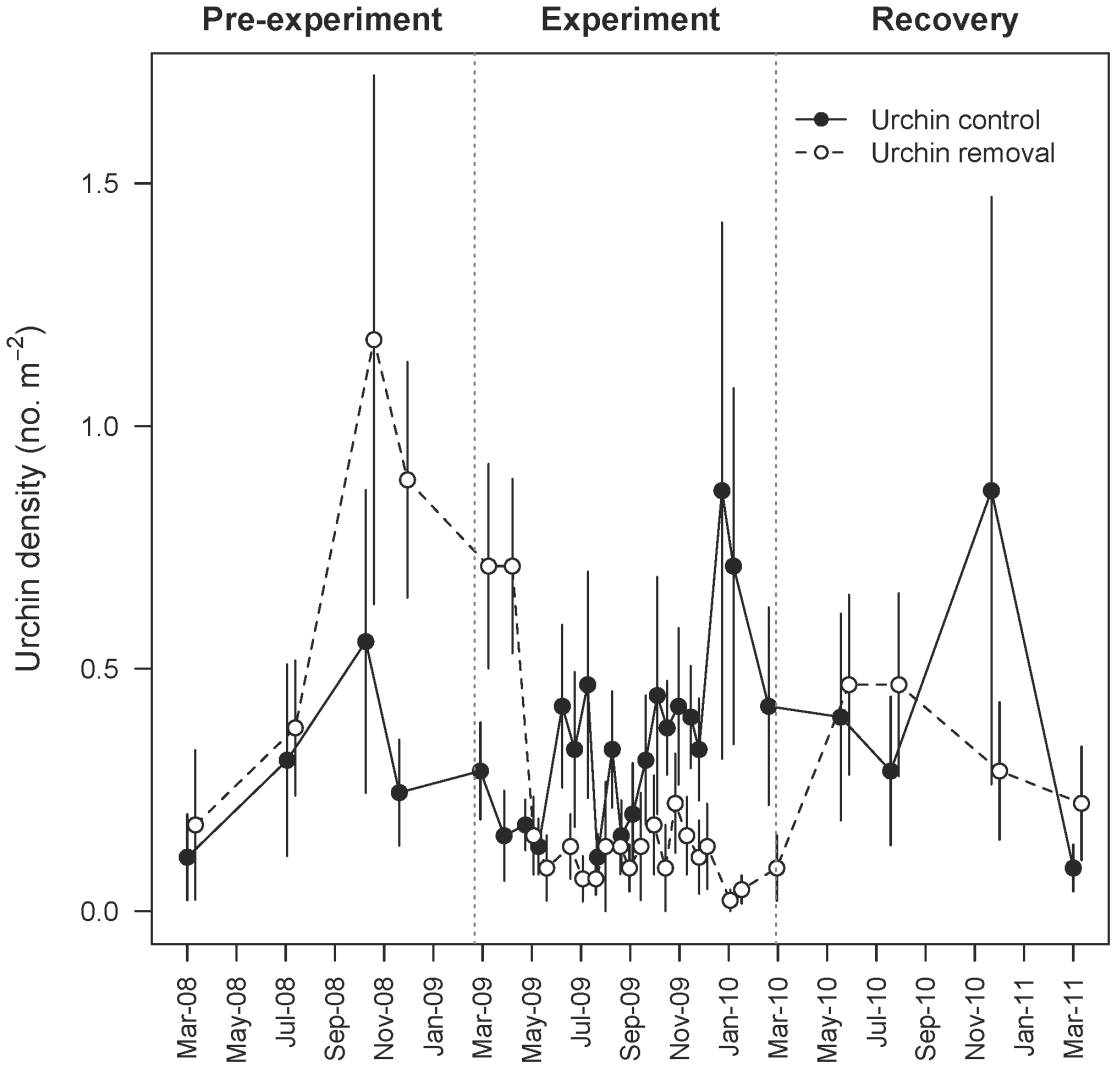
Densities of urchins (*Strongylocentrotus franciscanus*) before, during, and after the experiment. Variation in urchin densities (mean±SE; n = 9) during the experimental period was strongly dependent on urchin treatment (Table S1), with a sharp reduction observed on urchin removal transects and higher (but variable) densities on urchin control transects.

**Figure 2:**
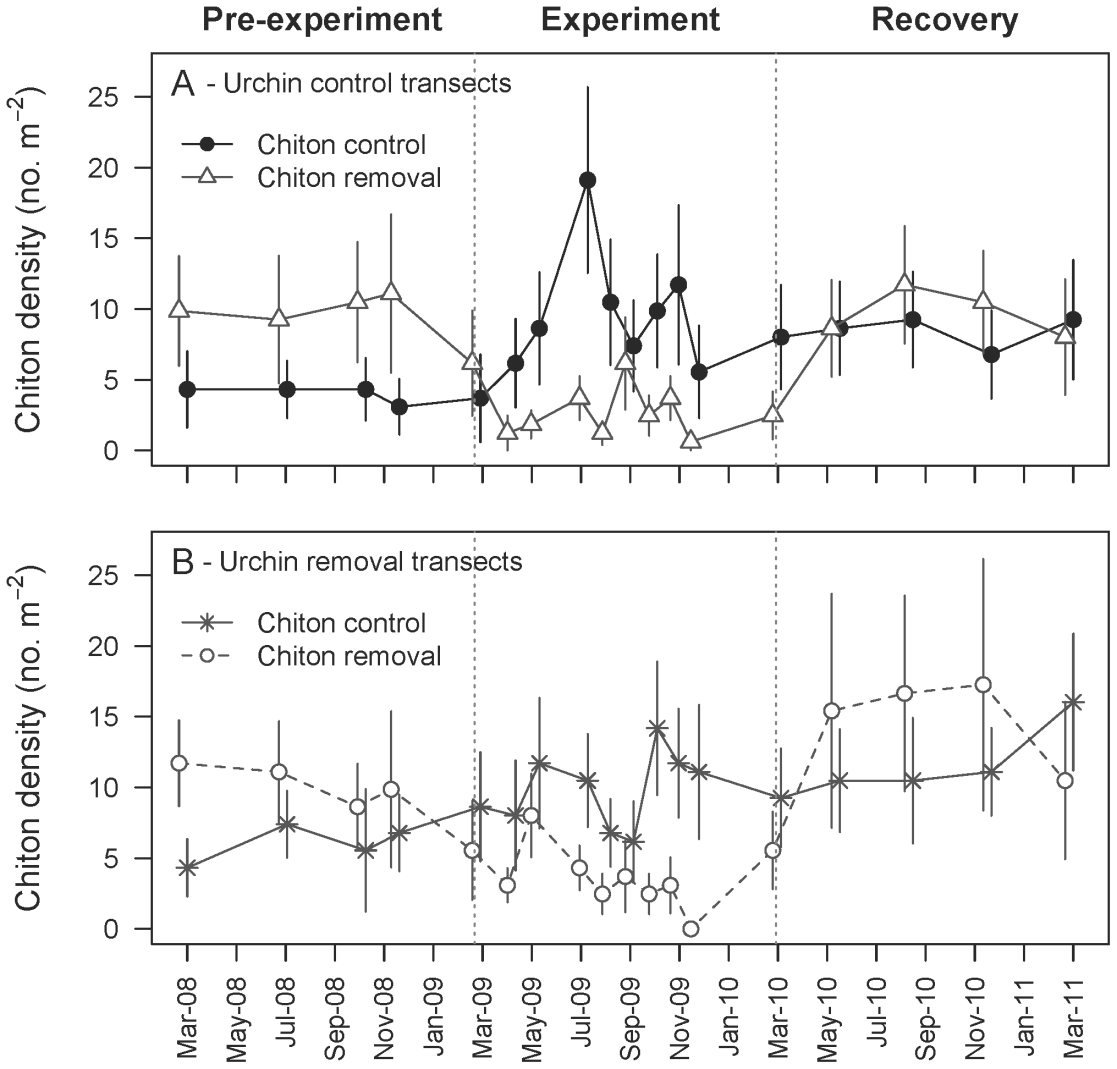
Densities of lined chitons (*Tonicella* spp.) before, during, and after the experiment. During the experimental period (March 2009–March 2010), chiton densities (mean±SE; n = 18) were reduced in the chiton removal treatment relative to the chiton control treatment. The best-fit model included the effects of chiton removal, time, and their interaction (Table S2).

We successfully reduced urchin (Fig. 1) and chiton (Fig. 2) densities in the removal treatments during the experimental period. Relative to the average urchin density before the experiment (March 2008–March 2009), 0.6±0.2 (mean±SE) urchins m^−2^ were removed from experimental transects, corresponding to an ∼84% reduction in urchins. Relative to the average chiton density prior to experimental removals, 5.8±1.5 chitons m^−2^ were removed from the experimental quadrats (a 61% reduction). We consider these estimates of reduction to be conservative, because it is unlikely that all of the urchins and chitons observed on experimental replicates two weeks after their removal had returned immediately. Based on the period when we were removing grazers (18 April 2009–24 January 2010), 0.12±0.05 urchins m^−2^ returned to removal transects every two weeks, which corresponds approximately to one urchin colonizing a removal transect per month. Likewise, we observed 3.6±0.5 chitons m^−2^ in experimental quadrats two weeks after their removal, which corresponds to ∼0.7 chitons colonizing a removal quadrat per month. In comparison, control quadrats harbored 9.7±1.6 chitons m^−2^ during the same removal period.

Urchin densities from March 2009– March 2010 were explained best by a model with an urchin term only (*w*_i = _0.91; Table S1). Graphically, a sharp reduction of urchins is evident on removal transects, but highly variable densities on control transects (Fig. 1). Chiton densities during the experimental period were explained best by a model including the effects of chiton, time, and their interaction (Table S2). The support for this model was not strong (*w*_i = _0.38) but the second best model was similar but lacked a chiton × time interaction term (*w*_i = _0.24, Δi = 0.91). Models lacking a chiton term received essentially no support (*w*_i_ <0.005; Table S2). Graphically, the chiton × time term reflects the different temporal trajectories of chiton densities in control versus removal plots (Fig. 2). The third best model (Δi <2) included an urchin term (Table S2), which may reflect the unexplained increase in chitons in control quadrats on urchin control transects in July 2009 (Fig. 2A). However, average chiton densities in unmanipulated quadrats during the experimental period were similar on urchin control (9.6±3.6 m^−2^; mean±SE) and urchin removal quadrats (9.6±3.0 m^−2^; mean±SE).

**Figure 3:**
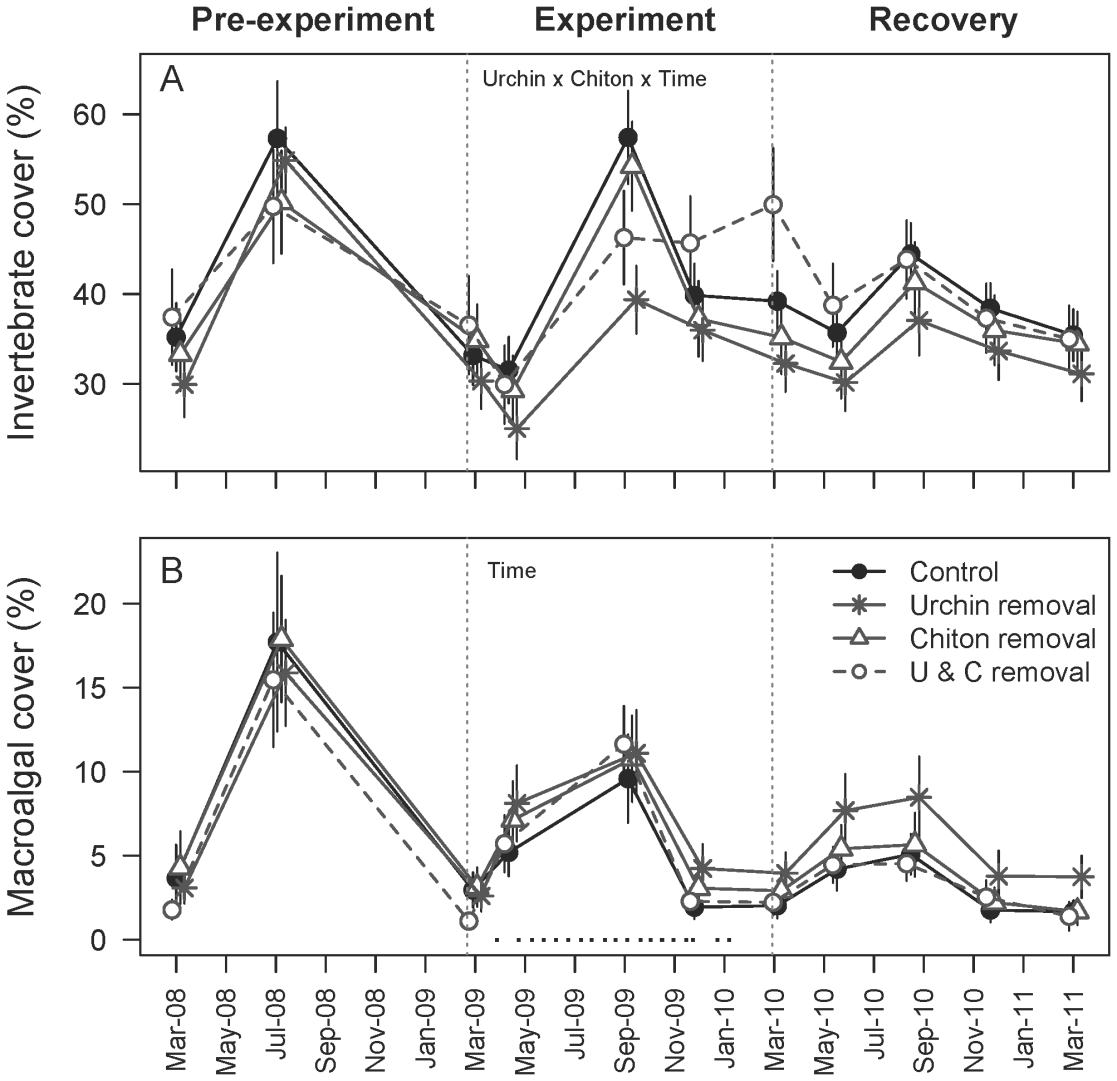
Percent cover of sessile invertebrates and macroalgae before, during, and after the experiment. The percent cover (mean±SE, n = 18) of invertebrates (A) during the experimental period (March 2009– March 2010) was variable, and depended on the interaction between urchin treatment, chiton treatment, and time (Table 1). Note that the percent cover of invertebrates was highest in quadrats subject to both urchin and chiton removal at the end of the experiment, but this effect was transient. After one year of recovery (March 2011), invertebrate cover was indistinguishable among experimental treatments after one year of recovery. In contrast, the percent cover of macroalgae (B) depended only on time. Black squares indicate the dates of consumer removal. The best model (Table 1) is indicated for each panel.

Invertebrate cover fluctuated greatly over time (Fig. 3A), and the complexity of the temporal response to experimental manipulation is supported statistically because the saturated model, including a three-way interaction, best fit the observed data (*w*_i = _0.54, Table 1). However, the second best model (*w*_i = _0.25, Δi = 1.51) included only urchin × chiton and urchin × time terms (in addition to the individual effects of urchin, chiton and time), suggesting that these interactions were most important in explaining variation in the data. Graphically, the urchin × chiton treatment interaction was apparent at the end of the experiment (March 2010), when the cover of sessile invertebrates in quadrats subjected to both urchin and chiton removal (hereafter referred to as the ‘double removal’ treatment) was 27–55% higher relative to the other three treatments (Fig. 3A). After one year of the removal of both grazers, invertebrate cover increased by 13% (a 37% relative increase, Fig. 3A). In comparison, invertebrate cover in control quadrats increased by 6% (an 18% relative increase), and the removal of either urchins or chitons resulted in minimal change (1–6% relative increase). The 13% increase in invertebrate cover observed in the double removal treatment was smaller than the observed temporal variability between March 2008 and September 2009 (20–30% fluctuations in cover), but similar to the fluctuations observed between May 2010 and March 2011. The urchin × time interaction can be visualized because invertebrate cover was similar among the four treatments during the beginning of the experiment (March – May 2009), but by September 2009 invertebrate cover had increased to a greater extent on urchin control transects (Fig. 3A).

Unlike epifauna, macroalgal cover did not respond strongly to grazer manipulations, but varied temporally during the experimental period (Table 1), peaking at the end of the summer (Fig. 3B). The best-fit model (*w*_i = _0.33) included only an effect of time, and models that lacked time received no support (Table 1). Three models that included the effects of urchin and chiton treatments received some support (*w*_i = _0.17–0.21, Δi <2), and are possibly related to the slightly lower percent cover of macroalgae on control plots (Fig. 3B). Macroalgal cover dropped to less than 5% during March, and increased to 10–20% cover between July and September (Fig. 3B). The cover of macroalgae was dominated primarily by rhodophytes, including the taxa *Callophyllis* spp., *Fryeella* spp., *Rhodymenia* spp., *Fauchea* spp., *Opuntiella californica*, and filamentous red algae.

**Figure 4:**
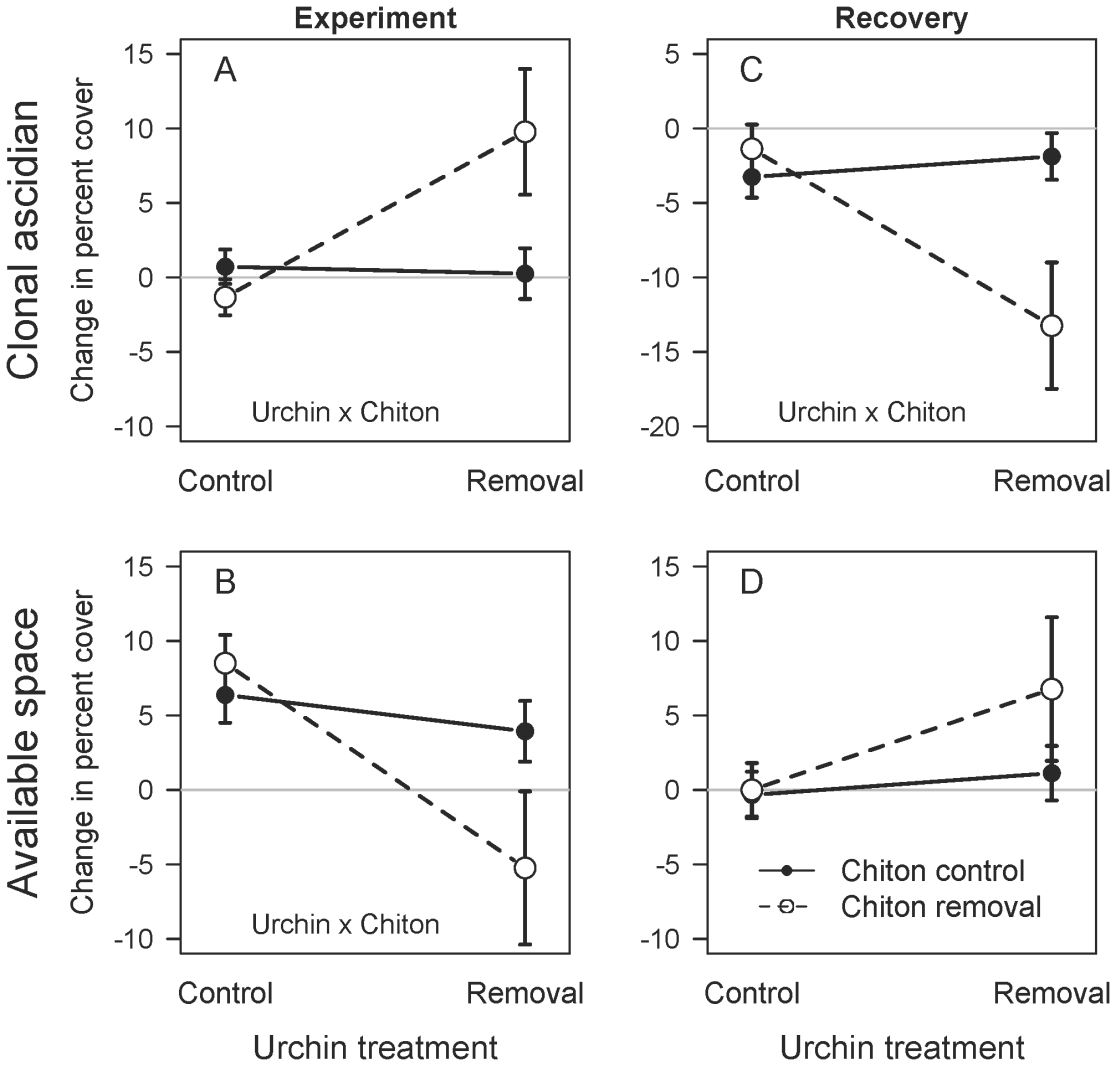
Change in percent cover of clonal ascidians and available space during and after the experiment. The experimental removal of both urchins and chitons caused an increase in the percent cover (mean±SE, n = 18) of clonal ascidians (A), and a decrease in available space (B) in quadrats. After a year of recovery, the percent cover of clonal ascidians (C) decreased in quadrats previously subjected to both urchin and chiton removal. In contrast, the effects of grazer removal did not affect the recovery of space (D). The best model (Table 2) is indicated for each panel, except when no candidate model was better than the null model.

The response of sessile invertebrates to grazer removal during the experimental period was driven primarily by clonal ascidians. Ascidian cover changed little, except when the removal of both grazers triggered a ∼10% increase in the cover of clonal ascidians (Fig. 4A), corresponding to a ∼75% change relative to initial starting conditions. Neither urchin nor chiton removal alone appeared to cause a change in the cover of clonal ascidians (Fig. 4A). The best-fit model (*w*_i = _0.81) included an urchin × chiton interaction (Table 2), supporting the visual non-additivity of the double removal treatment (Fig. 4A). Due to ascidian overgrowth, the amount of available space decreased by 5% in the double removal treatment, but increased by 4–9% in the other three treatments (Fig. 4B). Although the best-fit model (*w*_i = _0.44) for change in available space included an urchin × chiton interaction term, the second best model (*w*_i = _0.20) contained only an urchin treatment term, reflecting the overall decrease in available space in response to urchin removal (Fig. 4B). In summary, the removal of both grazers caused a larger change in the cover of ascidians and available space than expected based on the removal of each grazer in isolation (i.e., non-additivity). The most common clonal ascidian at the three sites, *Metandrocarpa taylori*, exhibited a 2.3 fold increase in percent cover in response to the removal of both grazers (Table S3).

When we stopped removing grazers, the densities of urchins (Fig. 1) and chitons (Fig. 2) recovered. Epifauna responded rapidly, with the total cover of sessile invertebrates in the double removal treatment dropping to levels comparable to the other treatments within approximately three months (June 2010; Fig. 3A). The cover of clonal ascidians dropped by ∼13% in the double removal treatment as compared with losses of only 1–3% in the other treatments (Fig. 4C), illustrating strong non-additivity (Table 2). In particular, *M. taylori* decreased in cover by 10% in the former treatment, a 69% relative drop (Table S3). The response of available space to the reintroduction of grazers during the recovery period was not as strong (Fig. 4D), because the null model received the most statistical support (*w*_i = _0.38).

## Discussion

Relative to temporal variability, the experimental removal of red urchins and lined chitons had small, but detectable, effects on epilithic communities. Notably, epifauna on subtidal rock walls did not respond to the manipulation of either grazer in isolation. Only the simultaneous removal of both grazers triggered a transient increase in cover of sessile invertebrates. Such non-additive effects have been observed in field experiments conducted in terrestrial [40], [41], freshwater [42], [43], and marine [44], [45] habitats. In our case, we evaluated the magnitude of the non-additive response in the context of three years of seasonal variation. Epifaunal cover fluctuated up to ∼30% on an intra-annual basis, in comparison with the ∼10% increase in cover of sessile invertebrates upon removal of both grazers. The small treatment effects may have occurred because our removals did not eliminate completely the grazers from exclusion areas. Alternatively, the absolute differences between treatments may have been biased by our decision to target relatively high densities of grazers for removal. Using our design, the average difference between control and removal replicates was likely smaller than if the treatments had been assigned at random. The relatively high invertebrate cover on urchin control transects midway through the experiment (September 2009) supports this hypothesis.

Although the non-additive increase in aggregate cover of invertebrates was relatively small, the increase was due primarily to clonal ascidians, which displayed a large (∼75%) relative increase in response to the removal of both grazers. Because our manipulations targeted relatively high densities of urchins and chitons, the change in cover within a quadrat was biased towards detecting a treatment effect (unlike the absolute cover among treatments). Therefore, we consider the observed change in ascidian cover to approach the maximum response possible to grazer release in these communities. The clonal growth strategy of ascidians is particularly well-suited to the rapid colonization of available space [31], but the experimental duration (∼9 months) was probably too short for slower growing epifauna (e.g., sponges) to respond to urchin removal. Indeed, a 27-month experimental removal of urchins from one rock wall resulted in significant changes to the relative abundances of several sessile taxa, including an increase in sponges, erect bryozoans, and ascidians [22].

Unlike epifauna, macroalgae did not respond to grazer removals. The absence of a macroalgal response may be due to light limitation on subtidal rock walls [26]. Sessile epifauna possess a competitive advantage relative to macroalgae in low-light environments [25], [27], [28], and thus the direct positive benefit of consumer removal on macroalgae may have been offset by the indirect negative effect of competition with ascidians. The observed intra-annual variation in algal cover is consistent with the hypothesis that photosynthesis and growth is limited in the winter by shorter day lengths and reduced light availability, but may also be caused by other covarying abiotic factors (e.g., temperature). The timing of the experiment did not favor algal growth because the grazer removals ended in January 2010, but macroalgal cover peaked between the summer months of July and September.

The weak benthic community response to urchin removal alone is surprising, because urchins are generally regarded to have strong impacts on sessile taxa [46]. However, in the San Juan Islands, the removal of red urchins does not result in changes to shallow (∼10 m depth) kelp communities [47]. Instead, the abundance of drift algae [48] may reduce active foraging and thus, benthic impacts of red urchins. Red urchins are relatively inactive, occur at low densities (<2 m^−2^), and tend not to form ‘feeding fronts’ at our study sites (R. E., personal observation) and other sites [47] in the San Juan Islands as observed in southern California [49]. Furthermore, red urchin populations had been harvested heavily in this region up until the fishery was closed in San Juan Channel in 1984 [50]. Therefore, the current and recent population densities may be lower than those historically and even a few decades ago. Despite urchin removal, Carter et al. [47] speculated that chitons and other mesograzers prevented the colonization of available substratum by kelp recruits. Our data support this hypothesis, but with respect to clonal ascidians on rock walls, rather than kelp on horizontal reefs. It is also possible that the epifaunal response to experimental removal was tempered by the presence of other mesograzers. Although red urchins and lined chitons are the most numerically abundant grazers on these rock walls [12], other chitons (e.g., *Mopalia* spp., *Lepidozona mertensii*) and echinoderms (e.g., *Henricia* spp.) may have limited the growth of sessile taxa.

In addition to the response of sessile taxa to grazer manipulation, the experimental design also permitted an examination of the numerical response of chitons to urchin removal. If urchins compete with chitons in this system, competitive release in the urchin removal treatments could result in an increase in chiton abundance. Alternatively, if chitons depend on urchins to continuously remove large invertebrate colonies to provide foraging space [12], urchin removal might result in a decline in a chiton abundance. Our data suggest neither; average chiton abundance was equivalent in unmanipulated plots on both urchin control and removal transects. However, it is still possible that the removal of urchins affected the behavior and/or grazing efficiency of the chitons already present in quadrats.

Less than 6 months after the cessation of removal treatments, the return of grazers into experimental transects and quadrats coincided with a decrease in the cover of clonal ascidians and an increase in available space. Urchin grazing likely reversed the temporary effects of the removal experiment, because red urchins eat established colonies of the clonal ascidian *Metandrocarpa taylori* [12]. This ascidian exhibited a pronounced increase, then decrease, in response to the removal and recovery of grazing pressure. The feeding activities of urchins thereby prevent the overgrowth of encrusting algae by ascidians and other epifauna. In addition, calcified crusts can benefit from the reduction in epiphytes afforded by mollusc grazing [51], [52], and *Tonicella* is known to be associated with and to consume calcified crusts [12], [16], [53]. It is unclear which algal taxa might benefit, because there is a diversity of calcified crusts in the San Juan Islands that respond differently to grazing [54]. Furthermore, there are four common species of *Tonicella* in our study system, which can be difficult to distinguish in the field. By removing all *Tonicella* in our experiment, we implicitly assumed that these species do not exhibit niche partitioning, an assumption that needs to be verified. Nevertheless, both chiton grazing and the inhibition of recruitment by some crustose corallines [33] can stabilize the persistence of an algal crust dominated state [19], [55], [56].

We suggest that *Tonicella* spp. and *Strongylocentrotus franciscanus* can be redundant components with respect to the maintenance of the limiting resource in this community, available space. The feeding behavior of these two grazers is similar, in that they both scrape rock surfaces using calcified jaw structures. Despite the redundancy observed in this study, red urchins and lined chitons cannot be considered equivalent in other respects. In particular, red urchins grow much larger than lined chitons, and size is well known to be a primary determinant of prey capture [57]. Whereas urchins are capable of clearing space through the consumption of macroalgae [46] and sessile invertebrates [12], [21], [22], small chitons (e.g., *Tonicella*) eat primarily microalgae and diatoms. Therefore, with respect to the removal of large invertebrate colonies and macroalgae, the size disparity between the two grazers is reflected as a functional difference. Intense urchin grazing is often responsible for the switch from a macroalgal-dominated community to a ‘barrens’, a state dominated by algal crusts [58]. Through grazing, urchins facilitate chitons and other mesograzers [12] by providing foraging space [59]. Our experiment supports the hypothesis that the indirect facilitation of chitons promotes the stability of algal crusts and bare rock on a small scale. The extent to which this mechanism scales up, both spatially and temporally, to influence the persistence of urchin ‘barrens’ remains an open question.

## Supporting Information

Table S1 Results of linear mixed effects models testing the fixed effects of urchin removal (U) and time (T) on log-transformed urchin density (no. m^−2^) measured on transects over the course of the one-year experimental period (March 2009– March 2010).
(DOCX)

Table S2 Results of linear mixed effects models testing the fixed effects of urchin removal (U), chiton removal (C), and time (T) on log-transformed chiton density (no. m^−2^) measured in quadrats over the course of the one-year experimental period (March 2009– March 2010).
(DOCX)

Table S3 Percent cover (mean±SE) of the three most common clonal ascidian species and the sum of other clonal ascidians in four experimental treatments.
(DOCX)

Dataset S1 Raw data used in this publication.
(XLSX)
