# Supplementary material for: Experimental Removal and Recovery of Subtidal Grazers Highlights the Importance of Functional Redundancy and Temporal Context
Source: PLoS One. 2013 Nov 8;8(11):e78969. doi: 10.1371/journal.pone.0078969 (PMC3826733; doi:10.1371/journal.pone.0078969)
Supplement: Table S1 — Results of linear mixed effects models testing the fixed effects of urchin removal (U) and time (T) on log-transformed urchin density (no. m−2) measured on transects over the course of the one-year experimental period (March 2009– March 2010). (DOCX) [file pone.0078969.s001.docx]

**Table S1**. Results of linear mixed effects models testing the fixed effects of urchin removal (U) and time (T) on log-transformed urchin density (no. m^-2^) measured on transects over the course of the one-year experimental period (March 2009 – March 2010).

| **Model** | ***K*** | **AIC_c_** | **Δi** | ***w*_i_** | **logLik** |
| --- | --- | --- | --- | --- | --- |
| *Urchin density (ln(x + 0.2))* |  |  |  |  |  |
| y ~ U × T (saturated model) | 39 | 533.29 | 26.84 | 0 | -222.15 |
| y ~ U + T | 22 | 517.63 | 11.18 | 0 | -235.13 |
| **y ~ U** | **5** | **506.44** | **0** | **0.91** | **-248.13** |
| y ~ T | 21 | 522.09 | 15.65 | 0 | -238.52 |
| y ~ 1 (null model) | 4 | 511.15 | 4.71 | 0.09 | -251.51 |

*K =* number of parameters; AIC_c_ = corrected AIC (AIC_c_); Δi = difference in AIC_c_ between the candidate model and the best model; *w*_i_ = Akaike weights; logLik = the log-likelihood (logLik). Candidate models with Δi < 2 are listed in bold.
