# Supplementary material for: Experimental Removal and Recovery of Subtidal Grazers Highlights the Importance of Functional Redundancy and Temporal Context
Source: PLoS One. 2013 Nov 8;8(11):e78969. doi: 10.1371/journal.pone.0078969 (PMC3826733; doi:10.1371/journal.pone.0078969)
Supplement: Table S2 — Results of linear mixed effects models testing the fixed effects of urchin removal (U), chiton removal (C), and time (T) on log-transformed chiton density (no. m−2) measured in quadrats over the course of the one-year experimental period (March 2009– March 2010). (DOCX) [file pone.0078969.s002.docx]

**Table S2**. Results of linear mixed effects models testing the fixed effects of urchin removal (U), chiton removal (C), and time (T) on log-transformed chiton density (no. m^-2^) measured in quadrats over the course of the one-year experimental period (March 2009 – March 2010).

| Model | *K* | AIC_c_ | Δi | *w*_i_ | logLik |
| --- | --- | --- | --- | --- | --- |
| *Chiton density (ln(x + 11.1))* |  |  |  |  |  |
| y ~ U × C × T (saturated model) | 44 | 710.94 | 25.41 | 0 | -308.54 |
| y ~ U + C + T + U:C + U:T + C:T | 35 | 699.26 | 13.73 | 0 | -312.79 |
| y ~ U + C + T + U:C + U:T | 26 | 699.85 | 14.33 | 0 | -322.91 |
| y ~ U + C + T + U:C + C:T | 26 | 689.36 | 3.84 | 0.056 | -317.67 |
| y ~ U + C + T + U:T + C:T | 34 | 697.05 | 11.52 | 0.001 | -312.79 |
| y ~ U + C + T + U:C | 17 | 690.17 | 4.64 | 0.038 | -327.65 |
| y ~ U + C + T + U:T | 25 | 697.70 | 12.18 | 0.001 | -322.91 |
| **y ~ U + C + T + C:T** | **25** | **687.21** | **1.69** | 0.165 | **-317.67** |
| y ~ U + C + U:C | 8 | 706.12 | 20.59 | 0 | -344.96 |
| y ~ U + T + U:T | 24 | 706.81 | 21.29 | 0 | -328.54 |
| **y ~ C + T + C:T** | **24** | **685.52** | **0.00** | 0.384 | **-317.90** |
| y ~ U + C + T | 16 | 688.07 | 2.55 | 0.107 | -327.65 |
| y ~ U + C | 7 | 704.07 | 18.55 | 0 | -344.96 |
| y ~ U + T | 15 | 697.23 | 11.71 | 0.001 | -333.28 |
| **y ~ C + T** | **15** | **686.43** | **0.91** | 0.243 | **-327.88** |
| y ~ U | 6 | 713.29 | 27.77 | 0 | -350.59 |
| y ~ C | 6 | 702.49 | 16.97 | 0 | -345.19 |
| y ~ T | 14 | 695.60 | 10.08 | 0.002 | -333.50 |
| y ~ 1 (null model) | 5 | 711.71 | 26.19 | 0 | -350.81 |

*K =* number of parameters; AIC_c_ = corrected AIC (AIC_c_); Δi = difference in AIC_c_ between the candidate model and the best model; *w*_i_ = Akaike weights; logLik = the log-likelihood (logLik). Candidate models with Δi < 2 are listed in bold.
