# Supplementary material for: Experimental Removal and Recovery of Subtidal Grazers Highlights the Importance of Functional Redundancy and Temporal Context
Source: PLoS One. 2013 Nov 8;8(11):e78969. doi: 10.1371/journal.pone.0078969 (PMC3826733; doi:10.1371/journal.pone.0078969)
Supplement: Table S3 — Percent cover (mean±SE) of the three most common clonal ascidian species and the sum of other clonal ascidians in four experimental treatments. (DOCX) [file pone.0078969.s003.docx]

**Table S3**. Percent cover (mean ± SE) of the three most common clonal ascidian species and the sum of other clonal ascidians in four experimental treatments.

| **Treatment** | **Period** | ***Metandrocarpa taylori*** | ***Didemnum carnulentum*** | ***Pycnoclavella stanleyi*** | **Other clonal ascidians** |
| --- | --- | --- | --- | --- | --- |
| Control | Pre-experiment | 3.5 ± 1.1 | 2.9 ± 1.2 | 1.9 ± 0.9 | 0.7 ± 0.3 |
| Control | Experiment | 6.4 ± 2.5 | 2 ± 0.7 | 1.1 ± 0.5 | 0.2 ± 0.1 |
| Control | Recovery | 2.6 ± 1 | 3.2 ± 1.3 | 0.6 ± 0.4 | 0.2 ± 0.1 |
|  |  |  |  |  |  |
| Chiton removal | Pre-experiment | 3.2 ± 1.6 | 4.1 ± 1.5 | 2.3 ± 1.1 | 0.9 ± 0.4 |
| Chiton removal | Experiment | 5.2 ± 2.4 | 2.6 ± 1 | 1.1 ± 0.6 | 0.3 ± 0.2 |
| Chiton removal | Recovery | 2.5 ± 1.2 | 4.4 ± 1.7 | 0.7 ± 0.4 | 0.2 ± 0.1 |
|  |  |  |  |  |  |
| Urchin removal | Pre-experiment | 4.5 ± 1.6 | 1.1 ± 0.5 | 1.7 ± 1.7 | 0.2 ± 0.1 |
| Urchin removal | Experiment | 6.1 ± 2.1 | 1.1 ± 0.5 | 0.5 ± 0.5 | 0.1 ± 0.1 |
| Urchin removal | Recovery | 3.1 ± 1.2 | 2.5 ± 1.3 | 0.3 ± 0.3 | 0.1 ± 0.1 |
|  |  |  |  |  |  |
| U & C removal | Pre-experiment | 6.8 ± 2.1 | 4.6 ± 2.1 | 1.5 ± 1.1 | 0.2 ± 0.2 |
| U & C removal | Experiment | 15.3 ± 5.2 | 5.4 ± 2.9 | 1.8 ± 1.3 | 0.3 ± 0.2 |
| U & C removal | Recovery | 4.8 ± 1.6 | 4 ± 2.3 | 0.7 ± 0.5 | 0.1 ± 0.1 |

Pre-experiment = March 2009, prior to consumer removal; Experiment = March 2010, after consumer removal; Recovery = March 2011, one year after the cessation of consumer removal
